# Population-based estimates of still birth, induced abortion and miscarriage in the Indian state of Bihar

**DOI:** 10.1186/s12884-014-0413-z

**Published:** 2014-12-17

**Authors:** Priyanka S Kochar, Rakhi Dandona, G Anil Kumar, Lalit Dandona

**Affiliations:** Public Health Foundation of India, Plot 47, Sector 44, Gurgaon, 122002 National Capital Region India; Institute for Health Metrics and Evaluation, University of Washington, Seattle, WA USA

**Keywords:** Abortion, Bihar, India, Miscarriage, Population-based, Still birth

## Abstract

**Background:**

We report population-based data on still birth, induced abortion and miscarriage from the Indian state of Bihar to assess the magnitude of the problem and to inform corrective action.

**Methods:**

A representative sample of women from all districts of Bihar with a pregnancy outcome in the last 12 months was obtained through multistage sampling in early 2012. Still birth rate was calculated as fetuses born with no sign of life at 7 or more months of gestation per 1,000 births. Induced abortion and miscarriage rates were defined as expulsion of dead fetuses at less than 7 months of gestation induced by any means or without inducement, respectively, per 1000 pregnancies that had an outcome. Multiple regression models were used to explore possible associations with stillbirths, induced abortions and miscarriages. Multi-level models were developed for the relatively less developed north zone and for the south zone of Bihar to examine contextual factors associated with still births, induced abortions and miscarriages.

**Results:**

Still birth rate was estimated as 20 per 1,000 births (95% CI 15.6-24.5), and induced abortion and miscarriage rates as 8.6 (6.6-10.6), and 46 (40.8-51.3) per 1,000 pregnancies with outcome, respectively. The odds of induced abortion and miscarriage were significantly higher in the south zone (odds ratio 2.53 [95% CI 1.79-3.57] and 1.27 [95% CI 1.10-1.47], respectively). In the multi-level model for the north zone, the odds of induced abortion were higher for women with husband’s having mean years of education higher than the state mean (2.62; 95% CI 1.47-4.69). Among the nine divisions of Bihar, comprising of groups of districts, higher induced abortion rate was associated with lower neonatal mortality rate (R^2^ = 0.68, p = 0.01).

**Conclusions:**

These population-based data show a significant burden of still births in Bihar, suggesting that addressing these must become an important part of maternal and child health initiatives. The higher induced abortion in the more developed districts, and the inverse trend between induced abortion and neonatal mortality rates, have programmatic implications.

**Electronic supplementary material:**

The online version of this article (doi:10.1186/s12884-014-0413-z) contains supplementary material, which is available to authorized users.

## Background

There is a dearth of reliable data on still birth, induced abortion and miscarriage in regions where these occur most frequently. Recent global estimates of still births suggest that an estimated 2.64 million babies were born dead in 2009 despite a 14.5% decline in the stillbirth rate between 1995 and 2009 [1,2]. This estimated trend in stillbirth rate reduction is slower than that for both under-5 child mortality and maternal mortality. The overwhelmingly large majority of still births still occur in south Asia and sub-Saharan Africa [2]. Global data on induced abortion incidence suggest that about one in five pregnancies ended in abortion in 2008 and that half of these abortions were unsafe [3]. The least available data relate to miscarriages, the incidence reported for which ranges between 8-50% [4,5].

Despite tremendous focus on maternal and new-born care in the past decade in India, pregnancy losses including still birth, induced abortion and miscarriage have not received much attention. Availability of reliable estimates and knowledge about causes of these outcomes are necessary for prevention of fetal loss as well as maternal deaths. In this background, we report population-based estimates and associations of still birth, induced abortion and miscarriage from the Indian state of Bihar which is the third most populous state in India [6]. These data were collected as part of the baseline household survey for the Ananya program evaluation. Ananya is a five-year program funded by Bill & Melinda Gates Foundation with the goal of reducing child and maternal mortality, fertility and under nutrition in Bihar [7]. We have recently reported neonatal mortality estimates and utilization of maternal care in Bihar state from the Ananya baseline household survey [8].

## Methods

The state of Bihar had a population of 104 million in 2011, with 11% urban [6]. This baseline survey is part of the Ananya evaluation, which has been approved by the institutional ethics committee of the Public Health Foundation of India and by the Health Ministry’s Screening Committee at the Indian Council of Medical Research. The survey design and sampling are described in the recently published report on neonatal mortality [8].

Briefly, using a multi-stage stratified random sampling approach, we obtained a representative sample of 1,017 clusters of about 75–150 households each from across all 38 districts of Bihar. This included 772 rural clusters and 245 urban clusters. The sample was based on having 15,390 live births in the sampled clusters which would give reasonable power to detect a significant change in the neonatal mortality rate over the intervention period [8].

Enumeration of the households in the sampled clusters was done to document all pregnancy outcomes in the last 12 months, including live births as well as still births, induced abortions and miscarriages in the last 12 months. A household was defined as people eating from the same kitchen. A detailed account of pregnancy outcomes in the last 12 months from the date of interview was assessed in each household. The preferred respondent for this information was the woman who had a pregnancy outcome in the last 12 months. In case this woman was not available, another woman of reproductive age in the household was asked for this information, and in the absence of this an older female in the household was asked. To ensure that no pregnancy outcome was missed during enumeration, we obtained data for the last 13 months from the date of enumeration but considered pregnancies with outcomes for the last 12 months for this analysis. Forty interviewers with experience in conducting health surveys were trained to use a structured questionnaire for confidential interviews in the local language to document all pregnancy outcomes in the last 12 months for the most recent pregnancy. This questionnaire is shown in Additional file 1. The data collection by interviewers was monitored by supervisors. At least three attempts were made to reach the households during the time period of data collection in a particular cluster which was generally three days. Data were collected from January to April 2012. Data were entered directly in a hand held device by the interviewers, which were scrutinized to detect and correct errors. About 30% of the data were collected by the interviewers under direct supervision and an additional 5% of the interviews were checked by the supervisors by visiting the respondent again. Data on these outcomes were also collected for women who had died due to pregnancy-related causes.

Any fetal loss either as still birth or induced abortion or miscarriage was carefully probed. Differentiation was attempted between the live newborn who had died soon after birth and stillbirths to avoid misclassification. After probing, if there was any evidence of life (breathing, crying at birth or definite movement of any voluntary muscles), the outcome was designated as a live birth [9]. Stillbirth was defined as fetus born with no sign of life at gestation period of 7 months or more. Induced abortion and miscarriage were distinguished by probing. Induced abortion was defined as expulsion of dead fetus at a gestation period of less than 7 months that was induced medically, surgically or by other means. Miscarriage was defined as expulsion of dead fetus at a gestation period of less than 7 months that occurred without any inducement. For each fetal loss, the duration of gestation at fetal loss was recorded, and the date of birth was recorded for live births. The sex of child/fetus expulsed was also recorded if known.

We report rates for still birth, induced abortion and miscarriage for the state of Bihar. Still birth rate was calculated as the number of still births per 1,000 births with gestation of 7 months or more, that were either live or still births [10]. Rates for induced abortions and miscarriages were calculated per 1,000 pregnancies that had a pregnancy outcome [10]. The estimates were reweighted for their probability of selection and non-response, and 95% confidence intervals (CI) are reported for the estimates. Sex ratio is reported as the number of female births for every 1,000 male births. STATA 11.2 (StataCorp, USA) software was used for analysis.

Multiple regression models were developed separately for the three outcomes: still birth, induced abortion and miscarriage in the last 12 months. The associations for these outcomes were explored with maternal age at birth, urban–rural residence, and north–south zones in the state of Bihar. The districts in Bihar were categorized into the relatively less developed north zone, which included 21 districts that are north of the Ganges river, and the south zone with the remaining 17 districts. The per capita GDP for the fiscal year 2009–10 was Indian Rupees (INR) 8,853 (US$ 187) for the north zone and INR 11,768 (US$ 248) for the south zone [11]. As sex was not known for the majority of the induced abortions and miscarriages, it was used only in the model for still birth.

We developed multi-level models separately for the north and south zones based on the results of multiple logistic regression to examine certain contextual factors associated with still birth, induced abortion and miscarriages. These models had maternal age at birth as the individual variable at level 1, nested within districts at level 2 in each zone. We considered the mean years of the highest education completed by fathers and by mothers of the children born in the last 12 months, the proportion of women who had received antenatal care (ANC) checkup within 3 months of pregnancy for the children born in the last 12 months, and proportion of population in the highest quartile of wealth index at the district level. These indicators for districts were aggregated from the data of the Ananya baseline household survey [8]. We also included the proportion of aggregated urban population at district level from the Census 2011 [6]. On examining the correlation between these indicators, the mean years of highest education completed by mothers and the proportion of population in the highest quartile of wealth index were highly correlated with the mean years of highest education completed by father. In addition, on comparing the drop in district level variance due to these three variables, the highest was by the mean years of highest education completed by father. Hence, only this variable was included in the final model. Each indicator included in the model was categorized into two categories - as “high” if the mean or proportion was higher than that of the state mean or proportion, and as “low” if the mean was lower than or equal to the state mean or proportion for that indicator. We then constructed an empty model or unconditional model without any exposure indicators to decompose the amount of variance that existed at the district level. Then, the maternal age at birth was included at individual level and later each district level indicator was added one by one to examine the changes in the district level variance separately for south and north zone. We report the change in variance at district level by each of the district level indicators considered. Finally, we ran a multi-level model with all the district level indicators together. The results of fixed and random effects are presented as odds ratios (OR) with 95% confidence interval (CI) and variance, respectively. Fixed effects account for all district-level variation by holding district constant, and random effects account for variability in the outcome across districts but not for covariates and allows estimates of variance at district levels. We also assessed the relationship of still births, induced abortions and miscarriages with neonatal mortality across the nine administrative divisions (groups of districts) of Bihar [8].

The checklist for how this manuscript followed the STROBE guidelines for reporting observational research is shown in Additional file 2.

## Results

Of the 116,784 eligible households, 110,094 (94.3%) participated – 4,405 (3.8%) were temporarily away from their household, 1,640 (1.4%) had no female respondent of any age, and 645 (0.5%) refused participation. The 110,094 participating households reported 16,062 cases of pregnancy outcomes in the last 12 months. Of these, for 15,138 (94.2%) cases the information on pregnancy outcome was provided by the women who had experienced that event in the last 12 months. The number of live births, still births, induced abortion and miscarriage in the last 12 months were 14,847, 283, 144, and 788, respectively. The estimated rate for still births in the state of Bihar was 20 per 1,000 births (95% CI 15.6-24.5), and for induced abortion and miscarriage was 8.6 (95% CI 6.6-10.6) and 46 (95% CI 40.8-51.3) per 1,000 pregnancies with outcome, respectively.

Considering the live births, sex ratio at birth was estimated to be 897 for Bihar, 895 for the north zone and 903 for the south zone. Among the 144 induced abortions reported, 111 (77.1%) fetuses were aborted within 3 months of pregnancy and 139 (96.5%) within 5 months of pregnancy. Of the 788 reported miscarriages, 551 (69.9%) occurred within 3 months of pregnancy and 738 (93.7%) within 5 months of pregnancy. Information on the sex of the fetus expelled was not available for 86.8% of the induced abortion and 84% of the miscarriage.

The rate of still births was similar in the districts in the north zone (20.2 per 1,000 births, 95% CI 13.8-26.6) and south zone (19.8 per 1,000 births, 95% CI 15.3-24.3) of Bihar. There was a significant difference in the rates of induced abortion and miscarriage in the north and south zone of Bihar (Table 1). The induced abortion rate in the south zone (15.5 per 1,000 pregnancies with outcome, 95% CI (11.3-19.7) was more than 3 times than that of north zone (4.7 per 1,000 pregnancies with outcome, 95% CI 2.9-6.6). Miscarriage rate was also higher in the south zone (p < 0.05).

**Table 1:** **Distribution of fetal outcomes for women who had a pregnancy outcome in the last 12 months in the Indian state of Bihar**

**Districts**	**Number in the last 12 months (% of total pregnancies with outcome)**				
	**Pregnancy with outcome**	**Live births**	**Still births**	**Induced abortion**	**Miscarriage**
	**(N)**	**N (%)**	**N (%)**	**N (%)**	**N (%)**
North Zone*	**9,029**	**8,436 (93.4)**	**145 (1.6)**	**49 (0.5)**	**399(4.4)**
Pashchim Champaran	731	674 (92.2)	12 (1.6)	2 (0.3)	43 (5.9)
Purba Champaran	714	651 (91.1)	13 (1.8)	1 (0.1)	49 (6.9)
Sheohar	148	138 (93.2)	3 (2)	0	7 (4.7)
Sitamarhi	398	375 (94.2)	7 (1.8)	1 (0.2)	15 (3.8)
Madhubani	476	446 (93.7)	12 (2.5)	0	18 (3.8)
Supaul	255	234 (91.8)	5 (2)	0	16 (6.3)
Araria	296	288 (97.3)	3 (1)	0	5 (1.7)
Kishanganj	250	238 (95.2)	3 (1.2)	1 (0.4)	8 (3.2)
Purnia	657	627 (95.4)	8 (1.2)	2 (0.3)	20 (3)
Katihar	498	456 (91.5)	11 (2.2)	5 (1)	26 (5.2)
Madhepura	272	258 (94.8)	3 (1.1)	1 (0.4)	10 (3.7)
Saharsa	253	241 (95.2)	4 (1.6)	1 (0.4)	7 (2.8)
Darbhanga	476	454 (95.4)	6 (1.3)	4 (0.8)	12 (2.5)
Muzaffarpur	764	718 (94)	12 (1.6)	4 (0.5)	30 (3.9)
Vaishali	412	375 (91)	8 (1.9)	8 (2)	21 (5.1)
Samastipur	358	346 (96.7)	3 (0.8)	3 (0.8)	6 (1.7)
Begusarai	335	322 (96.1)	3 (0.9)	2 (0.6)	8 (2.4)
Khagaria	152	143 (94.1)	3 (2)	1 (0.7)	5 (3.3)
Gopalganj	548	507 (92.5)	11 (2)	4 (0.7)	26 (4.7)
Siwan	471	427 (90.7)	3 (0.7)	4 (0.8)	37 (7.9)
Saran	565	518 (91.7)	12 (2.1)	5 (0.9)	30 (5.3)
South Zone*	**7,033**	**6,411 (91.2)**	**138 (2.0)**	**95 (1.4)**	**389 (5.5)**
Bhagalpur	571	538 (94.2)	11 (1.9)	3 (0.5)	19 (3.3)
Banka	485	452 (93.2)	9 (1.9	5 (1)	19 (4.0)
Munger	275	254 (92.4)	5 (1.9)	4 (1.4)	12 (4.4)
Lakhisarai	220	204 (92.7)	2 (0.9)	2 (0.9)	12 (5.4)
Sheikhpura	182	172 (94.5)	3 (1.6)	1 (0.5)	6 (3.3)
Nalanda	540	473 (87.6)	13 (2.4)	16 (3)	38 (7)
Patna	981	861 (87.8)	17 (1.7)	18 (1.8)	85 (8.7)
Bhojpur	575	519 (90.3)	15 (2.6)	11 (1.9)	30 (5.2)
Buxar	292	256 (87.7)	2 (0.7)	4 (1.4)	30 (10.3)
Kaimur (Bhabua)	320	288 (90)	2 (0.6)	5 (1.6)	25 (7.8)
Rohtas	471	430 (91.3)	16 (3.4)	5 (1)	20 (4.2)
Aurangabad	179	169 (94.4)	3 (1.7)	0	7 (3.9)
Gaya	374	349 (93.3)	10 (2.7)	2 (0.5)	13 (3.5)
Nawada	591	557 (94.2)	12 (2)	2 (0.3)	20 (3.4)
Jamui	350	320 (91.4)	4 (1.1)	7 (2)	19 (5.4)
Jehanabad	518	468 (90.3)	14 (2.7)	9 (1.7)	27 (5.2)
Arwal	109	101 (92.7)	0	1 (0.9)	7 (6.4)
**Overall**	**16,062**	**14,847 (92.4)**	**283 (1.8)**	**144 (0.9)**	**788 (4.9)**

*North zone includes districts that are north of Ganga. The south zone has districts lying to the south of Ganga river.

With multiple logistic regression, the only significant association with still births was slightly higher odds for males than for females (Table 2). Multiple logistic regression model for induced abortion revealed higher odds with increasing age of the mother (Table 3). As compared with the north zone, in the south zone the odds of induced abortion (OR 2.53, 95% CI 1.79-3.57) and miscarriages (OR 1.27, 95% CI 1.10-1.47) were significantly higher.

**Table 2:** **Associations of still births in the last 12 months in the Indian state of Bihar using the multiple logistic regressions**

**Variable**	**Categories**		**Still births in the last 12 months**	
		**Total (N = 15,130)**	**Number (%)**	**Odds Ratio (95% CI)**
Maternal age at birth (years)	Up to 29	11,647	210 (1.8)	1.00
	30 or more	3,483	73 (2.1)	1.17 (0.89-1.54)
Residence	Rural	12,374	242 (1.9)	1.00
	Urban	2,756	41 (1.5)	0.78 (0.56-1.09)
Zone of Bihar	North	8,581	145 (1.7)	1.00
	South	6,549	138 (2.1)	1.20 (0.95-1.53)
Sex*	Female	7,192	166 (2.3)	1.00
	Male	7,916	110 (1.4)	1.38 (1.08-1.76)

*Data missing: sex for 22.

CI denotes confidence interval.

**Table 3:** **Associations of induced abortion and miscarriage in the last 12 months in the Indian state of Bihar using the multiple logistic regressions**

**Variable**	**Categories**		**Induced abortion in the last 12 months**		**Miscarriage in the last 12 months**	
		**Total (N = 16,062)**	**Number (%)**	**Odds Ratio (95% CI)**	**Number (%)**	**Odds Ratio (95% CI)**
Maternal age at birth (years)	Up to 29	12,319	88 (0.7)	1.00	584 (4.7)	1.00
	30 or more	3,743	56 (1.5)	2.17 (1.55-3.04)	204 (5.5)	1.16 (0.99-1.37)
Residence	Rural	13,134	113 (0.9)	1.00	647 (4.9)	1.00
	Urban	2,928	31 (1.1)	1.25 (0.84-1.87)	141 (4.8)	0.98 (0.81-1.18)
Zone of Bihar	North	9,029	49 (0.5)	1.00	399 (4.4)	1.00
	South	7,033	95 (1.3)	2.53 (1.79-3.57)	389 (5.5)	1.27 (1.10-1.47)

CI denotes confidence interval.

No significant associations were found in the multi-level models for still birth and miscarriage (data not shown). In the multi-level model with induced abortion as an outcome for the north and south zones of Bihar, maternal age at birth was associated with a higher odds of induced abortion in both the north zone (OR 2.36; 95% CI 1.33-4.19) and the south zone (OR 2.13; 95% CI 1.40-3.24) (Table 4). In the north zone, the odds of induced abortion were significantly higher in districts with mean years of father’s education higher than the state mean (OR 2.62; 95% CI 1.47-4.69). The highest reduction in variance in the model for the north zone was also observed with this variable, with the variance dropping from 0.214 in the null model to 0.000. The multilevel model for the south zone did not reveal any significant associations.

**Table 4:** **Multi-level regression model for the relationship of district level indicators with induced abortion in the last 12 months in the Indian state of Bihar**

		**North Zone**				**South Zone**			
**Variable**	**Category**	**Total**	**Abortion**			**Total**	**Abortion**		
		**N = 9,029**		**Odds of having Abortion**	Variance*	**N = 7,033**		**Odds of having Abortion**	Variance*
		**N (%)**	**N (%)**	**(95% CI)**	(SE)**	**N (%)**	**N (%)**	**(95% CI)**	(SE)**
**Null model**					**0.214 (0.03)**				**0.161 (0.03)**
Maternal age at birth (years)	Up to 29	6,877 (76.2)	20 (0.3)	1.00	0.259 (0.05)	5,442 (77.4)	59 (1.1)	1.00	0.155 (0.03)
	30 or more	2,152 (23.8)	20 (0.9)	2.36 (1.33-4.19)		1,591 (22.6)	36 (2.3)	2.13 (1.40-3.24)	
Districts based on mean years of the highest education completed by father†	Lower or equal to state mean	6,304 (69.8)	23 (0.4)	1.00	0.000 (0.00)	1,945 (27.7)	33 (1.7)	1.00	0.125 (0.02)
	Higher than state mean	2,725 (30.2)	26 (1.0)	2.62 (1.47-4.69)		5,088 (72.3)	62 (1.2)	0.93 (0.50-1.73)	
Districts based on proportion of women received ANC check-up within 3 months of pregnancy‡	Lower than state proportion	4,030 (44.6)	15 (0.4)	1.00	0.161 (0.02)	2,619 (37.2)	26 (1.0)	1.00	0.106 (0.02)
	Higher than state proportion	4,999 (55.4)	34 (0.7)	1.72 (0.93-3.21)		4,414 (62.8)	69 (1.6)	1.50 (0.80-2.82)	
Districts based on proportion of urban population¶	Lower than state proportion	8,694 (96.3)	47 (0.5)	1.00	0.260 (0.05)	2,326 (33.1)	24 (1.0)	1.00	0.120 (0.02)
	Higher than state proportion	335 (3.7)	2 (0.6)	1.38 (0.32-6.04)		4,707 (66.9)	71(1.5)	1.31 (0.74-2.30)	

*The variance here will signify the distribution of random effects and the variability accounted by adding indicator separately.

†State mean years of the highest education completed by father were 5.5 years.

‡ANC denotes antenatal checkup; State proportion for women who had received ANC care was 44%.

¶The state proportion for urban population was11.3%.

**SE denotes standard error.

CI denotes confidence interval.

**Figure 1:**
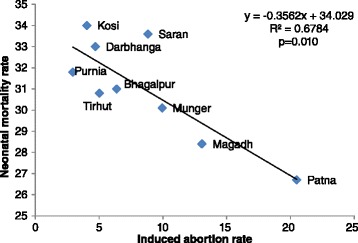
**Relationship between induced abortion rate and neonatal mortality rate across the administrative divisions in the Indian state of Bihar.**

A significant inverse linear relationship was found between the induced abortion rate and neonatal mortality rate across the administrative divisions of Bihar (Figure 1); the divisions with higher induced abortion rates had lower neonatal mortality (p = 0.01). No significant relationship was observed for still birth and miscarriage with neonatal mortality (data not shown). Also, no significant association was observed between sex ratio at birth and the induced abortion or miscarriage rate across the administrative divisions of Bihar.

## Discussion

We report data on still birth, induced abortion and miscarriage from a representative sample of pregnant women from across all districts in Bihar. Reliable information on the magnitude and associations of these pregnancy outcomes can inform interventions to reduce such outcomes. An important aspect of these data is that we probed signs of evidence of life in the fetus and the gestational age to attempt accurate classification of still births, induced abortions and miscarriages.

The rate of still birth in Bihar in our study was 20 per 1,000 births for the year 2011, as data were collected in early 2012 for pregnancy outcomes in the past 12 months. Globally, only 2% of all still births are counted through the vital registration system, and therefore the global estimates largely depend on household surveys and modeling [12]. Issues of under-reporting and misclassification in documentation of still births are known in household surveys, which are the main source of data on still births in countries with the highest burden [12]. Considering the most recent household level data available for India from the District Level Household Survey-3 (DLHS-3) of 2007–08, the documentation of still birth was based on recall of pregnancies in the last 3 years [13]. The estimated still birth rate for Bihar was 18.8 per 1,000 births from DLHS-3 of 2007–08 [13]. Even though the estimates of still birth rate are similar between our survey and DLHS-3 that was done 4–5 years prior to our survey, these rates are not comparable. The documentation of still births and neonatal deaths in DLHS-3 was based on the respondent’s assessment without probing questions about signs of life in the newborn as was done in our study. In addition, neither gestational age of the fetus nor duration of pregnancy was recorded in DLHS-3. In the absence of gestational age, birth weight is often used as a proxy indicator of gestational age to identify still birth (late fetal death). However, birth weight data are often poorly reported in surveys in developing countries [14,15]. In our survey, 50% of the children born in the last 12 months were reported by the mothers to have not been weighed at all at birth, and only 26% were weighed on the day they were born. Birth weight was not documented in DLHS-3 of 2007–08. Another source reporting still birth estimates is the Sample Registration System (SRS) in India. The estimate for still birth from SRS for the state of Bihar was 1 per 1,000 total births in the year 2011, highlighting gross underestimation in these data [16]. A limitation for our study was that because the population studied was relatively illiterate and unable to recall the exact weeks of gestation, we could only use months of gestation for calculation of rates. Still birth was therefore defined as birth of a dead fetus with gestation period of 7 months or more. This is slightly more than the 28 weeks gestation period usually used for defining still births, so we may have slightly underestimated the magnitude of stillbirths in our study.

We did not find any significant relationship between still births and the assessed variables of interest either at the individual or district levels. The rate of still birth has been reported to be associated with indices of sex equality including education, reproductive control, access to antenatal care, maternal age, and parity [17,18]. Due to the non-availability of all these data in the enumeration done in our study, we were unable to assess these associations. Another limitation of our data was that we could not distinguish between intrapartum still births (occurring during labor) and antepartum still births. Documentation of the timing of still births relative to the delivery or skin condition of the expelled fetus can assist with such classification in surveys [12,18]. Such information is important from health services perspective as 25-60% of intrapartum still births are avoidable with improved obstetric care and improved response to intrapartum complications [12,19-21]. Of importance to note is that still births are not included in the Millennium Development Goals whereas child deaths are, and certain socio-cultural factors around still births make it a hidden phenomenon at the societal level [22]. Under the National Rural Health Mission, significant efforts are being made in India to strengthen the neonatal services by establishment of Comprehensive/Basic Emergency Obstetric and Neonatal Care Centers and also to increase institutional deliveries under the safe motherhood interventions [23-25]. Efforts to improve availability of data on still births in household surveys and in health facilities, which allow differentiation between intrapartum and antepartum still births, would be an important step to improve evidence-based prevention of still births and to improve maternal care in India [19]. In addition, inclusion of standard perinatal death certificate in developing country settings, and improved documentation of still birth in verbal autopsies can also further build evidence related to still births [12,18].

Data on incidence of induced abortions and miscarriages is important to monitor progress in maternal health and access to family planning. The estimated incidence of induced abortions and miscarriages for Bihar in our study was 8.6 and 46 per 1,000 pregnancies with outcome in the last 12 months, respectively. The reported rates for induced abortion and miscarriage in DLHS-3 for Bihar were 9 and 41 per 1,000 pregnancies based on recall of pregnancies in the last 3 years [13]. More recently the Annual Health Survey has reported that 5% of pregnancies in women aged 15–49 years resulted in abortion (which included induced and spontaneous loss of fetus) in Bihar during reference years 2008–10 [26]. This is similar to the total of 54.6 induced abortions and miscarriages per 1,000 pregnancies with outcome in our study.

Our estimate of miscarriage rate could be an underestimate as many miscarriages go unnoticed early in pregnancy. In addition, some women may also be not comfortable in reporting miscarriage as it is may have adverse social implications. On the other hand, some induced abortions may be reported as miscarriages because women who underwent abortion for prenatal sex selection related to preference for boys, which is common in India [27,28] may not want to report these abortions. As we did not have data on outcome or sex of children for pregnancies prior to the last 12 months in our study, we are unable to directly comment on the contribution of sex preference to induced abortion in our study. However, it is likely that the relatively low sex ratio at birth of 897 that we found in Bihar is influenced by female feticide, as the sex ratio at birth expected for humans is 942, i.e. a ratio of 0.485 for female births to total births [29]. SRS has reported a sex ratio at birth of 908 for India in 2012, which included Bihar with a sex ratio of 909 and some other states of such as Haryana, Jammu and Kashmir, Maharashtra, Punjab, Rajasthan, and Uttar Pradesh with sex ratios below 900 [16]. The south zone of Bihar with higher induced abortion rate did not have a lower sex ratio at birth, so there was no evidence in our study of a major contribution of female feticide to the differential between the abortion rates between the north and south zones of Bihar.

The lower neonatal mortality rate in divisions with higher induced abortion rate that we found in our study indicates that aborting unwanted pregnancies may be leading to better neonatal outcomes. We found a strong association of induced abortion with higher maternal age in our survey, which may be related to more at-risk pregnancies in older women and with higher likelihood of older women opting for abortion in case of unwanted pregnancy.

We found significantly higher rates for both induced abortion and miscarriage in the south zone of the state as compared with the north zone. Given the south zone is socioeconomically more developed than its counterpart, availability and access to health facilities for maternal care is higher, and hence likely higher chance of availability of options for sex selective abortion [11]. No significant associations of contextual factors were found for the south zone. In the north zone, father’s education was significantly associated with the likelihood of abortion. Previous studies have reported an increased likelihood of the educated parents to have information about and access to abortion services as compared with the non-educated parents [30-33]. These data point to the need to target men as well to improve maternal health.

## Conclusions

These population-based data from all districts of the Indian state of Bihar show a significant burden of stillbirths and provide some insights into induced abortions and miscarriages. Improved and reliable data are crucial to ensure that stillbirths, induced abortions and miscarriages get relevant attention in health planning and policy. To make still births count as a public health concern commensurate with its burden, issues around under-reporting and misclassification of still births need to be addressed, and the lack of information on the causes of still births at sub-national levels in India has to be addressed for effective prevention and intervention programmes.

## Additional files

Additional file 1:
**Survey questionnaire.**
Additional file 2:
**STROBE checklist of items for reports of cross-sectional studies.**

## Abbreviations

ANC: Antenatal care
CI: Confidence interval
DLHS: District Level Household Survey
GDP: Gross Domestic Product
INR: Indian Rupees
OR: Odds ratio
SRS: Sample Registration System
