# Supplementary material for: Population-based estimates of still birth, induced abortion and miscarriage in the Indian state of Bihar
Source: BMC Pregnancy Childbirth. 2014 Dec 17;14:413. doi: 10.1186/s12884-014-0413-z (PMC4300052; doi:10.1186/s12884-014-0413-z)
Supplement: Additional file 1: — Survey questionnaire. [file 12884_2014_413_MOESM1_ESM.docx]

**Population-based estimates of still birth, induced abortion and miscarriage in the Indian state of Bihar**

Priyanka S Kochar, Rakhi Dandona, G Anil Kumar, Lalit Dandona

**Additional file 1: Survey questionnaire**

| S.NO. | PARTICULAR | Name | CODE | S.NO. | PARTICULAR | RESPONSE | CODE |
| --- | --- | --- | --- | --- | --- | --- | --- |
| A1 | District | _________ |  | A7 | Unique Reference ID |  |  |
| A2 | Block | _________ |  | A8 | Name of Household Head | ____________ |  |
| A3 | Village/UFS | _________ |  | A9 | Caste |  |  |
| A4 | Structure Type. | 1=Residential  2=Non-residential |  | A10 | Result of visit | [Proceed to Q1 only] |  |
| A5 | Structure No |  |  |  |  |  |  |
| A6 | Household No. |  |  | A11 | Date | DD/MM/YYYY | |
| A12 | Investigator ID |  |  |  | | | |

| **Caste Codes**--- 1=Brahmin, 2=Rajput/Thakur, 3=Bhumihar 4=Kayasth/ Srivastava/Lala, 5=Dalit 6=Chamar, 7=Dusadh/Paswan, 8=Musahar 9=Pasi, 10=Dhobi, 11=Bhuiya, 12=Chaupal, 13=Bantar, 14=Rajwar, 15=Yadav, 16=Vaishya/Bania, 17=Kurmi, 18=Shah, 19=Muslim, 88=Other Caste Or Tribe (Specify) |
| --- |
| **Results Code**--1= Complete, 2= Refused, 3= HH Migrated, 4= HH temporarily away, 5= No female respondent of age present to respond |

| 1 | Are there any women/girls in this household between the ages of 12 and 60 years? | | | | | Yes | 1 | | Proceed to 3.1 | |
| --- | --- | --- | --- | --- | --- | --- | --- | --- | --- | --- |
|  |  |  |  |  |  | No | 2 | | Proceed to 2 | |
| 2 | Was there any other woman/girl who died during pregnancy, at the time of delivery or within 45 days of giving birth? | | | | | Yes | 1 | | Proceed to 3.1 | |
|  |  |  |  |  |  | No | 2 | | End | |
| 3.1 | Please tell me the name of each woman/girl in the household between the ages of 12 and 60 years and whether she was ever married. | | | | | | | | | |
| A. Line No. | B. Name of woman/girl  STARTING WITH THE OLDEST WOMAN TO THE YOUNGEST WOMAN | C. Age (in completed years) | D. Whether ever married?  Yes -1  No -2 | E. Identity of the respondent  Self-1  Other-2 | F. In the time since [MONTH]* last year, did [WOMAN] gave birth to a child (alive or dead) or had an abortion or miscarriage?  *MONTH CAN BE ACCORDING LOCAL CALENDAR, BUT SHOULD INCLUDE AT LEAST ONE FULL YEAR FROM THE DATE OF INTERVIEW  IF MULTIPLE EVENTS TOOK PLACE, LIST EACH EVENT ON A SEPARATE LINE | | | | | |
|  |  |  |  |  | Gave birth to alive child | | | 1 | | Go to Q3.2A |
|  |  |  |  |  | Gave birth to dead child | | | 2 | |  |
|  |  |  |  |  | Had abortion | | | 3 | | Go to Q 3.2A |
|  |  |  |  |  | Had miscarriage | | | 4 | |  |
|  |  |  |  |  | Not Applicable | | | 5 | | GOTO NEXT WOMAN |
| (1) |  |  |  |  |  | | | | | |
| (2) |  |  |  |  |  | | | | | |

| In addition to the women/girls mentioned above, was there any other woman/girl who died during pregnancy, at the time of delivery or within 45 days of giving birth? | | | | | | | |
| --- | --- | --- | --- | --- | --- | --- | --- |
| 3.2A | If yes, record her cause of death and time of death and proceed | Reason_______________ _ | | | | | |
|  |  | ____/______/_________  DD MM YYYY | | | | | |
| 3.2B | Name of woman who gave birth to a child (alive or dead) or had abortion or had miscarriage since [MONTH] last year, and line number of event: | | | | A. Name:___________  B. Line Number from Q2A | | |
|  | For every birth alive or dead (Response 1 or 2 to Q4), proceed to Q4  For every abortion or miscarriage (Response 3 or 4 to Q15), complete Q15-Q17 | | | | | | |
| 4 | Did the child/expulsed fetus cry at birth? | | Yes | | 1 |  | |
|  |  |  | No | | 2 |  | |
|  |  |  | Don’t know | | 3 |  | |
| 5 | Was the child/expulsed fetus breathing after birth? | | Yes | | 1 |  | |
|  |  |  | No | | 2 |  | |
|  |  |  | Don’t know | | 3 |  | |
| 6 | Did the child/expulsed fetus make any movement after birth? | | Yes | | 1 |  | |
|  |  |  | No | | 2 |  | |
|  |  |  | Don’t know | | 3 |  | |
|  | INTERVIEWER:  Pregnancy Outcome  If the response is YES to any of Q4-6, interpretation is that the child was born ALIVE;  If response is NO to ALL three Q4-6, interpretation is that the child was born DEAD;  If response is NO to some of Q4-6 and DK to some, or DK to all, further information is needed (Q7) to determine if the child was born alive or dead | | Child born ALIVE | | 1 | Go to Q9 | |
|  |  |  | Child born DEAD | | 2 | Go to Q15 | |
|  |  |  | Further information needed | | 3 | Go to Q7 | |
| 7 | Did the doctor/nurse/dai/other health provider tell you that the child was alive or dead? | | ALIVE | | 1 | Go to Q9 | |
|  |  |  | DEAD | | 2 | Go to Q15 | |
|  |  |  | Did not tell or Don’t know | | 3 | Go to Q8 | |
| 8 | If response to Q7 is 3, ask the respondent if the child was born alive or dead | | ALIVE | | 1 | Go to 9 | |
|  |  |  | DEAD | | 2 | Go to Q15 | |
| 9 | When was the child born?  Interviewer use local event calendar to ascertain this to the nearest month, and record the month and year.  Please also record the day of birth, if it is known. | | | _ _  DD  _ _  MM  _ _ _ _  YYYY  Number of months ago = | | |  |
| 10 | If child was born alive, is the child alive now? | | Yes | | 1 | Go to Q11 | |
|  |  |  | No | | 2 | Go to Q 13 | |
|  |  |  | Don’t know | | 3 | Go to Q 11 | |
| 11 | What is the name of the child? | | __________________________ | |  |  | |
| 12 | What is the gender of the child? | | Female | | 1 | END | |
|  |  |  | Male | | 2 |  |  |
|  |  |  | Don’t know | | 3 |  |  |
| 13 | If answer to Q10 is NO, for how long after birth was the child alive?  Record days if less than one month, or in months if one month or more. | | In days  / In months | |  |  | |
| 14 | What was the gender of the child? | | Female | | 1 | END | |
|  |  |  | Male | | 2 |  |  |
|  |  |  | Don’t know | | 3 |  |  |
| 15 | When did this abortion, miscarriage, or dead birth take place?  Interviewer use local event calendar to ascertain this to the nearest month, and record the month and year.  Please also record the day of the abortion/ miscarriage/ dead birth, if it is known. | | _ _ /_ _ / _ _  DD MM YY  Number of months ago= | |  |  | |
| 16 | If child was born dead or if there was an abortion or miscarriage, interviewer assess the completed gestational age in months using local event calendar. | | Completed Months | |  | If more than 6 months in case of abortion or miscarriage, then go to 4 | |
| 17 | What was the gender of the child? | | Female | | 1 | END | |
|  |  |  | Male | | 2 |  |  |
|  |  |  | Don’t know | | 3 |  |  |
