# Supplementary material for: Population-based estimates of still birth, induced abortion and miscarriage in the Indian state of Bihar
Source: BMC Pregnancy Childbirth. 2014 Dec 17;14:413. doi: 10.1186/s12884-014-0413-z (PMC4300052; doi:10.1186/s12884-014-0413-z)
Supplement: Additional file 2: — STROBE checklist of items for reports of cross-sectional studies. [file 12884_2014_413_MOESM2_ESM.doc]

**Population-based estimates of still birth, induced abortion and miscarriage in the Indian state of Bihar**

Priyanka S Kochar, Rakhi Dandona, G Anil Kumar, Lalit Dandona

**Additional file 2: STROBE checklist of items for reports of cross-sectional studies**

**Title and abstract**

**Item No 1(*a*):** Indicate the study’s design with a commonly used term in the title or the abstract

**Response:** We have included the term population-based estimates in the title and the abstract.

**Item No 1(*b*):** Provide in the abstract an informative and balanced summary of what was done and what was found

**Response:** We have described the summary of what was done and what was found in the methods and results sections of the abstract.

**Introduction**

Background/rationale

**Item No 2**: Explain the scientific background and rationale for the investigation being reported

**Response:** These are mentioned in the background section.

Objectives

**Item No 3**: State specific objectives, including any pre-specified hypotheses

**Response:** The objective for the paper is mentioned in the background section (last paragraph): “In this background, we report population-based estimates and associations of still birth, induced abortion and miscarriage from the Indian state of Bihar which is the third most populous state in India.” There was no pre-specified hypothesis for this paper.

**Methods**

Study design

**Item No 4**: Present key elements of study design early in the paper

**Response:** The study design is described in the methods section (paragraphs 1-2).

Setting

**Item No 5**: Describe the setting, locations, and relevant dates, including periods of recruitment, exposure, follow-up, and data collection

**Response:** This information is provided in the methods section (paragraphs 1-4).

Participants

**Item No 6**: (*a*) Give the eligibility criteria, and the sources and methods of selection of participants

**Response:** This information is provided in the methods section (paragraphs 2-4).

.

Variables

**Item No 7**: Clearly define all outcomes, exposures, predictors, potential confounders, and effect modifiers. Give diagnostic criteria, if applicable

**Response:** This information is provided in the methods section (paragraphs 5-7).

Data sources/ measurement

**Item No 8**: For each variable of interest, give sources of data and details of methods of assessment (measurement). Describe comparability of assessment methods if there is more than one group

**Response:** This information is provided in the methods section (paragraphs 6-7).

Bias

**Item No 9**: Describe any efforts to address potential sources of bias

**Response:** The efforts to reduce potential bias through obtaining a representative population-based sample and supervision of data collection are described in the methods section (paragraphs 1 and 4).

Study size

**Item No 10**: Explain how the study size was arrived at

**Response:** This information is provided in the methods section (paragraphs 1-2).

Quantitative variables

**Item No 11**: Explain how quantitative variables were handled in the analyses. If applicable, describe which groupings were chosen and why

**Response:** This is explained in the methods section (paragraphs 5-7).

Statistical methods

**Item No 12 (*a*):** Describe all statistical methods, including those used to control for confounding

**Response:** This is explained in the methods section (paragraphs 6-7).

**Item No 12 (*b*):** Describe any methods used to examine subgroups and interactions

**Response:** We examined effects of contextual factors on pregnancy outcomes by the north and south zones. This information is described in the methods section (paragraph 7).

**Item No 12 (*c*):** Explain how missing data were addressed

**Response:** The proportion of missing data is very low in this study, the details of which are provided in the footnote for Table 2.

**Item No 12 (*d*):** If applicable, describe analytical methods taking account of sampling strategy

**Response:** This is explained in the methods section (paragraph 5).

**Item No 12 (e):** Describe any sensitivity analyses

**Response:** We did not perform any sensitivity analyses as it was not relevant for our findings.

**Results**

**Item No 13 (a):** Report numbers of individuals at each stage of study—eg numbers potentially eligible, examined for eligibility, confirmed eligible, included in the study, completing follow-up, and analysed

**Response:** This is described in the results section (paragraphs 1-2).

**Item No 13 (b):** Give reasons for non-participation at each stage

**Response:** This is mentioned in the results section (paragraph 1).

**Item No 13 (c):** Consider use of a flow diagram

**Response:** We believe that details of participation presented in the results section (paragraph 1) are adequate to convey the participation details, and that a flow diagram is not needed.

Descriptive data

**Item No 14 (*a*):** Give characteristics of study participants (eg demographic, clinical, social) and information on exposures and potential confounders

**Response:** This information is provided in Table 1, 2 and 3.

**Item No 14 (*b*):** Indicate number of participants with missing data for each variable of interest

**Response:** This is mentioned in the footnote for Table 2.

Outcome data

**Item No 15:** Report numbers of outcome events or summary measures

**Response:** This information is reported in the results section (paragraph 1- 2).

Main results

**Item No 16 (*a*):** Give unadjusted estimates and, if applicable, confounder-adjusted estimates and their precision (eg, 95% confidence interval). Make clear which confounders were adjusted for and why they were included

**Response:** This information is provided in the results section (paragraphs 3-5) and Tables 2-4.

**Item No 16 (*b*):** Report category boundaries when continuous variables were categorized

**Response:** Category boundaries of continuous variables are given in Table 2-4.

**Item No 16 (*c*):** If relevant, consider translating estimates of relative risk into absolute risk for a meaningful time period

**Response:** This is not relevant for this analysis.

Other analyses

**Item No 17:** Report other analyses done—eg analyses of subgroups and interactions, and sensitivity analyses

**Response:** Differences in estimates by north and south zones are described in the results section (paragraphs 3-5). We did not perform any sensitivity analyses as it was not relevant for our findings.

**Discussion**

Key results

**Item No 18:** Summarise key results with reference to study objectives

**Response:** This information is provided in the discussion section (first paragraph).

Limitations

**Item No 19:** Discuss limitations of the study, taking into account sources of potential bias or imprecision. Discuss both direction and magnitude of any potential bias

**Response:** This is discussed in the discussion section (paragraph 2-3).

Interpretation

**Item No 20:** Give a cautious overall interpretation of results considering objectives, limitations, multiplicity of analyses, results from similar studies, and other relevant evidence

**Response:** This is done in the discussion section.

Generalizability

**Item No 21:** Discuss the generalizability (external validity) of the study results

**Response:** This is discussed in the discussion section.

**Other information**

Funding

**Item No 22:** Give the source of funding and the role of the funders for the present study and, if applicable, for the original study on which the present article is based

**Response:** The source of funding is mentioned in the acknowledgements section. The design of the study and interpretations of the findings in this paper benefited from discussions with the funding agency, but the views expressed in this paper are those of the authors and do not necessarily reflect the views of the funding agency.
